# Harnessing single-cell and multi-omics insights: STING pathway-based predictive signature for immunotherapy response in lung adenocarcinoma

**DOI:** 10.3389/fimmu.2025.1575084

**Published:** 2025-04-16

**Authors:** Yang Ding, Dingli Wang, Dali Yan, Jun Fan, Zongli Ding, Lei Xue

**Affiliations:** ^1^ Department of Pathology, Nanjing Drum Tower Hospital Group Suqian Hospital, Suqian, China; ^2^ Department of Lung Cancer, Tianjin Lung Cancer Center, National Clinical Research Center for Cancer, Key Laboratory of Cancer Prevention and Therapy, Tianjin’s Clinical Research Center for Cancer, Tianjin Medical University Cancer Institute and Hospital, Tianjin, China; ^3^ Department of Oncology, The Affiliated Huai’an Hospital of Xuzhou Medical University and the Second People’s Hospital of Huai’an, Huai’an, China; ^4^ Department of Thoracic Surgery, The First Affiliated Hospital with Nanjing Medical University, Nanjing, China; ^5^ Department of Geriatrics, The Affiliated Huai’an Hospital of Xuzhou Medical University and the Second People’s Hospital of Huai’an, Huai’an, China

**Keywords:** non-small cell lung cancer, lung adenocarcinoma, single-cell RNA sequencing, prognosis signature, machine learning

## Abstract

**Background:**

Lung adenocarcinoma is the most prevalent type of small-cell carcinoma, with a poor prognosis. For advanced-stage patients, the efficacy of immunotherapy is suboptimal. The STING signaling pathway plays a pivotal role in the immunotherapy of lung adenocarcinoma; therefore, further investigation into the relationship between the STING pathway and lung adenocarcinoma is warranted.

**Methods:**

We conducted a comprehensive analysis integrating single-cell RNA sequencing (scRNA-seq) data with bulk transcriptomic profiles from public databases (GEO, TCGA). STING pathway-related genes were identified through Genecard database. Advanced bioinformatics analyses using R packages (Seurat, CellChat) revealed transcriptomic heterogeneity, intercellular communication networks, and immune landscape characteristics. We developed a STING pathway-related signature (STINGsig) using 101 machine learning frameworks. The functional significance of ERRFI1, a key component of STINGsig, was validated through mouse models and multicolor flow cytometry, particularly examining its role in enhancing antitumor immunity and potential synergy with α-PD1 therapy.

**Results:**

Our single-cell analysis identified and characterized 15 distinct cell populations, including epithelial cells, macrophages, fibroblasts, T cells, B cells, and endothelial cells, each with unique marker gene profiles. STING pathway activity scoring revealed elevated activation in neutrophils, epithelial cells, B cells, and T cells, contrasting with lower activity in inflammatory macrophages. Cell-cell communication analysis demonstrated enhanced interaction networks in high-STING-score cells, particularly evident in fibroblasts and endothelial cells. The developed STINGsig showed robust prognostic value and revealed distinct immune microenvironment characteristics between risk groups. Notably, ERRFI1 knockdown experiments confirmed its significant role in modulating antitumor immunity and enhancing α-PD1 therapy response.

**Conclusion:**

The STING-related pathway exhibited distinct expression levels across 15 cell populations, with high-score cells showing enhanced tumor-promoting pathways, active immune interactions, and enrichment in fibroblasts and IFI27+ inflammatory macrophages. In contrast, low-score cells were associated with epithelial phenotypes and reduced immune activity. We developed a robust STING pathway-related signature (STINGsig), which identified key prognostic genes and was linked to the immune microenvironment. Through *in vivo* experiments, we confirmed that knockdown of ERRFI1, a critical gene within the STINGsig, significantly enhances antitumor immunity and synergizes with α-PD1 therapy in a lung cancer model, underscoring its therapeutic potential in modulating immune responses.

## 1 Introduction

Lung cancer is the most prevalent form of cancer within the respiratory system and represent the leading cause of cancer-related mortality among both genders (1–3). Based on histological classification, lung cancer can be divided into non-small cell lung cancer (NSCLC) and small cell lung cancer (SCLC), with NSCLC accounting for over 80% of lung cancer cases (4, 5). NSCLC primarily consists of adenocarcinoma (LUAD) and squamous cell carcinoma (LUSC), with lung adenocarcinoma being the most common histological type of NSCLC, comprising approximately 40% of all lung cancer cases (6–8). Currently, for early-stage lung adenocarcinoma, surgical intervention remains the most effective therapeutic approach (9). However, for patients with advanced lung adenocarcinoma, platinum-based adjuvant chemotherapy has shown limited efficacy in improving prognosis, with a 5-year survival rate of only 5% (10). The emergence of immune checkpoint inhibitors has significantly improved clinical outcomes for patients, although prognosis remains suboptimal (11–13). This can be attributed to the highly heterogeneous nature of LUAD (14, 15). Therefore, there is an urgent need to elucidate the underlying mechanisms contributing to poor prognosis in lung adenocarcinoma patients and to develop predictive scoring models for immune therapy outcomes, thereby providing personalized treatment strategies for these patients.

The emergence of immunotherapy, particularly immune checkpoint inhibitors (ICIs) (16–20), has brought revolutionary breakthroughs in the treatment of lung adenocarcinoma. PD-1/PD-L1 inhibitors, which block key pathways through which tumor cells evade immune surveillance and reactivate the body’s antitumor immune response, have become a crucial treatment option for advanced NSCLC. However, clinical practice has shown that only approximately 20-30% of patients demonstrate durable responses to immune checkpoint inhibitor therapy, with most patients developing primary or acquired resistance (21, 22). This heterogeneity in treatment response has prompted researchers to explore biomarkers and signaling pathways that can predict immunotherapy efficacy and improve treatment efficiency (23). STING (Stimulator of Interferon Genes) is a critical innate immune signaling pathway responsible for detecting endogenous and exogenous DNA damage and triggering immune protective responses within the body, while also playing a vital role in maintaining tissue homeostasis (24). The STING pathway plays an essential role in cancer biology (25). Abnormal DNA fragments in tumor cells can activate the STING pathway, thereby promoting dendritic cell maturation and enhancing their functionality, which in turn facilitates T-cell-mediated tumor eradication (26). Previous studies have indicated that rocaglamide can activate the cGAS-STING pathway in non-small cell lung cancer, thereby enhancing the cytotoxicity of NK cells against tumor cells (27). Inhibition of DNA damage response and subsequent activation of the STING pathway can induce the activation of cytotoxic T lymphocytes (28). Recently, the STING signaling pathway has garnered increasing attention in the context of lung adenocarcinoma research, with the potential to improve the prognosis of patients undergoing immune therapy (29–31). Therefore, a more comprehensive understanding of the relationship between immune therapy and the STING signaling pathway in lung adenocarcinoma is essential.

With advancements in high-throughput sequencing techniques, particularly single-cell RNA sequencing (scRNA-seq), researchers can now explore the complex mechanisms underlying lung adenocarcinoma with unprecedented resolution (32). This study focuses on the role of the STING signaling pathway in lung adenocarcinoma, aiming to investigate its contribution to tumorigenesis and progression. Through bioinformatic analysis of publicly available data, the study seeks to identify potential pathways associated with the STING signaling pathway that could improve prognosis and provide valuable insights for the treatment of lung adenocarcinoma patients. Additionally, our *in vivo* experiments confirmed that knockdown of ERRFI1, a key gene within the STING signature (STINGsig), significantly enhances antitumor immunity and synergizes with α-PD1 therapy in a lung cancer model. Compared to monotherapy or control groups, the combination of shERRFI1 and α-PD1 treatment markedly reduced tumor burden in mice. Kaplan-Meier survival analysis revealed that the combination therapy conferred the greatest survival benefit, significantly prolonging survival compared to either monotherapy or control groups. Furthermore, the combination treatment significantly increased the frequencies of TNF-α+ and GZMB+ CD8+ T cells, indicating enhanced activation of cytotoxic T cells.

## 2 Methods

### 2.1 Preparation and preprocessing of single-cell RNA sequencing data

We conducted an extensive analysis of single-cell RNA sequencing data from 11 lung adenocarcinoma samples within the HRA005794 dataset. Utilizing the Seurat R package (33), we implemented an integrated computational workflow for single-cell transcriptomic data analysis. The expression data were normalized using the LogNormalize method, with a scaling factor set at 10,000. Subsequently, 2,000 highly variable genes were identified, and the expression intensities of these genes were preprocessed before performing principal component analysis (PCA). Batch effect correction was systematically applied using the Harmony R package (34). Our analytical strategy combined the advanced functionalities of Seurat and Harmony, including NormalizeData, FindVariableFeatures, ScaleData, RunPCA, FindNeighbors, FindClusters, and RunUMAP, to achieve comprehensive molecular characterization (35).

### 2.2 Global RNA sequencing data analysis

Transcriptomic data and clinical annotations for lung adenocarcinoma were obtained from The Cancer Genome Atlas (TCGA) database (https://portal.gdc.cancer.gov), encompassing extensive RNA sequencing data, mutation landscapes, and long-term survival information. To enhance model validation, six independent cohorts were supplemented from the Gene Expression Omnibus (GEO) database (http://www.ncbi.nlm.nih.gov/geo).

To standardize data preprocessing, all datasets underwent logarithmic transformation. Batch effects were mitigated using the combat function from the sva R package, ensuring analytical consistency and minimizing technical variation.

### 2.3 Investigation of potential mechanisms and pathways

To elucidate underlying mechanisms, we performed Gene Set Variation Analysis (GSVA) for a comprehensive exploration of potential biological processes (36). Our analytical approach leveraged curated gene sets derived from the Molecular Signatures Database (MSigDB), facilitating an extensive pathway analysis.

### 2.4 Cell-cell communication

We integrated gene expression profiles to systematically dissect cell-cell communication dynamics using the CellChat framework (37). The CellChatDB ligand-receptor database served as our reference framework, and the established CellChat analysis protocol was strictly adhered to. Through meticulous computational analysis, we identified cell-type-specific interactions and detected ligands and receptors highly expressed in distinct cell populations. This methodology allowed for precise inference of intercellular communication networks, with a particular focus on ligand-receptor interactions exhibiting elevated expression.

### 2.5 Construction of prognostic signatures

A comprehensive univariate Cox regression analysis was performed to systematically assess the prognostic impact of individual genes in lung adenocarcinoma patients. Using a rigorous 10-fold cross-validation approach, we evaluated 101 distinct machine learning algorithm combinations encompassing a broad range of predictive methods, including stepwise Cox regression, Lasso, Ridge, partial least squares Cox regression (plsRcox), CoxBoost, random survival forests (RSF), generalized boosted regression modeling (GBM), elastic net (Enet), supervised principal components (SuperPC), and survival support vector machines (survival-SVM). The primary objective was to construct a robust STING pathway-related signature (STINGsig) and quantify its optimal predictive performance using the highest concordance index (C-index). To validate the diagnostic accuracy of the signature, we conducted comprehensive assessments using receiver operating characteristic (ROC) curve analysis and multidimensional principal component analysis (PCA).

### 2.6 Cell culture

The murine lung cancer cell line M109 was obtained from the American Type Culture Collection (ATCC). The cells were cultured in high-glucose Dulbecco’s Modified Eagle Medium (DMEM) supplemented with 10% fetal bovine serum (FBS) and 1% penicillin-streptomycin (P/S). Cultures were maintained in a humidified incubator at 37°C with 5% CO_2_.

### 2.7 shRNA knockdown cell line construction

To generate M109 cells with ERRF1 knockdown, lentiviral particles were produced by co-transfecting HEK293T cells with the ERRF1 shRNA plasmid (targeting sequence: 5’-GGACAUCAAGAAGCUGUUA-3’)), packaging plasmid (psPAX2), and envelope plasmid (pMD2.G). Viral supernatants were harvested after 48 hours, filtered, and used to transduce M109 cells in the presence of polybrene (8 µg/mL). Stable M109-Luc-ERRF1 knockdown cells were selected using puromycin (2 µg/mL) for 7–10 days, and knockdown efficiency was validated by qPCR and/or Western blot analysis.

### 2.8 Lung cancer model construction and IVIS imaging

M109-Luc cells (luciferase-labeled) were injected into the tail vein of 6–8-week-old Balb/c mice (3 × 10^5 cells per mouse) to establish a lung cancer model. Tumor development was monitored weekly using an IVIS Lumina III imaging system. Mice were intraperitoneally injected with D-luciferin (150 mg/kg) 10 minutes before imaging to ensure maximum luminescence signal. Luciferase activity was quantified as photons/second/cm2/steradian (p/s/cm2/sr) using Living Image software. Tumor growth was analyzed over time, and histopathological confirmation of lung metastases was performed at study endpoint.

### 2.9 Flow cytometry analysis of lung tumor tissue

Fresh lung tumor tissue was minced and enzymatically digested using collagenase IV (1 mg/mL) and DNase I (0.1 mg/mL) at 37°C for 30 minutes. The resulting cell suspension was filtered to obtain a single-cell suspension, followed by red blood cell lysis and resuspension in flow cytometry staining buffer. To block Fc receptors, cells were incubated with anti-CD16/32 antibody at 4°C for 10 minutes. Subsequently, cells were stained with a viability dye (e.g., Zombie Aqua or PI) at 4°C for 15 minutes in the dark to exclude dead cells. After washing with PBS, surface staining was performed by incubating cells with anti-CD45 (APC-A750), anti-CD3(PC7) and anti-CD8 (FITC) antibodies at 4°C for 30 minutes in the dark. Following fixation and permeabilization, intracellular staining was carried out using anti-GZMB (PerCP-Cy5.5) and anti-TNF-α (ECD) antibodies at 4°C for 30 minutes in the dark. Flow cytometry analysis was conducted with the following gating strategy: firstly, lymphocytes were gated based on forward scatter (FSC) and side scatter (SSC); secondly, live cells were gated within the viability dye-negative population; thirdly, CD45+ leukocytes were gated within the CD45-positive population. From this, CD8+ T cells were further identified based on CD8 positivity, and the proportions of GZMB+ and TNFA+ subsets were analyzed. Data were processed using FlowJo software to calculate the percentages of each subset and their fluorescence intensities, followed by statistical analysis.

### 2.10 Statistical analysis

All data processing, statistical analysis, and visualization were performed using R software (version 4.2.0). Survival characteristics of molecular subtypes were rigorously evaluated through Kaplan-Meier survival analysis and Log-rank statistical tests. Comparative analysis of continuous variables between groups was performed using appropriate parametric tests (t-test) or non-parametric tests (Wilcoxon), while associations between categorical variables were systematically assessed via chi-square or Fisher’s exact tests. To mitigate the issue of multiple testing, p-values were adjusted using the false discovery rate (FDR) method. Inter-variable relationships were explored through Pearson correlation analysis. All statistical tests were two-tailed, with statistical significance defined as p < 0.05.

## 3 Results

### 3.1 Sample integration, cell clustering, and annotation

**Figure 1:**
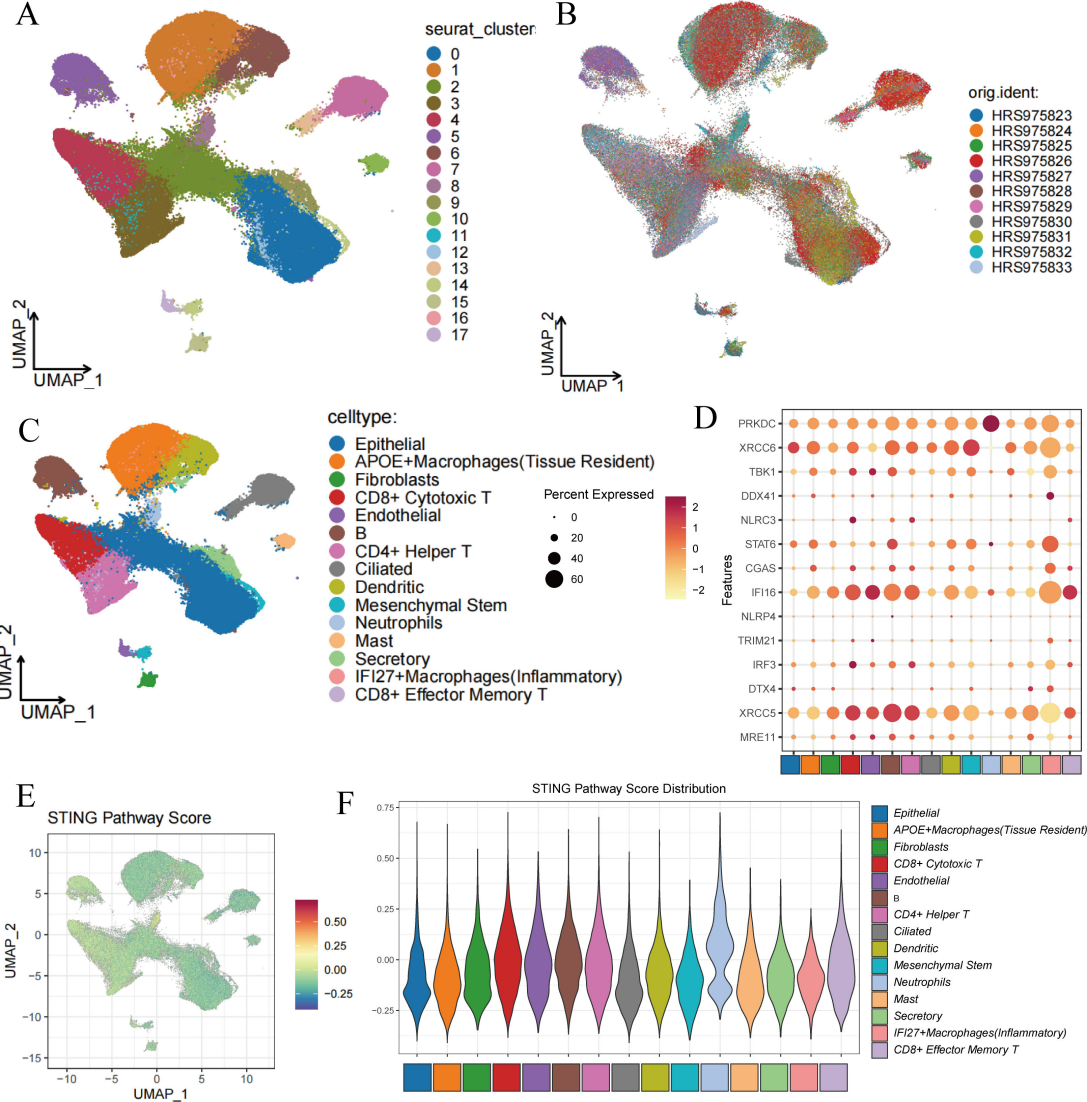
Cell clustering and score of STING Pathway. **(A)** Cell clustering by Seurat. **(B)** Integration of 11 samples from lung adenocarcinoma. **(C)** Annotation of cell clusters. **(D)** The expression of 14 marker genes in cell clusters. **(E)** The distribution of high-score cells and low-score cells. **(F)** Scores of STING Pathway distribution in clusters.

This study utilized 11 single-cell RNA sequencing samples from the HRA005794 dataset, all derived from human lung cancer tissues. Initially, Seurat was employed to identify 18 distinct cell clusters within these 11 sequencing samples (**Figure 1A**). Subsequently, the Harmony algorithm was applied to integrate these samples into a unified dataset (**Figure 1B**). To better characterize these clusters, the expression of 18 commonly recognized marker genes was examined, including SFTPC, MRC1, SCGB3A2, IL7R, GZMB, MS4A1, HLA-DQB1, DNAH12, S100A8, SCGB1A1, TPSB2, CD3D, NAPSA, RSPH1, CCDC26, DCN, MARCO, and VWF.

Marker genes for type II alveolar epithelial cells, such as SFTPC and NAPSA, were predominantly expressed in clusters 0, 10, and 12; while epithelial markers SCGB3A2 and SCGB1A1 were mainly expressed in clusters 0, 2, 4, 9, 12, and 13. The endothelial marker VWF was primarily observed in cluster 17, and fibroblast marker DCN was mainly expressed in cluster 15. Monocyte marker S100A8 showed elevated expression in clusters 1, 6, and 8, whereas macrophage markers MRC1 and MARCO were expressed in clusters 0, 1, 2, 6, 7, 13, and 14. Mast cell marker TPSB2 was predominantly found in cluster 10. Ciliated cell markers DNAH12 and RSPH1 were primarily expressed in clusters 7, 13, and 14. For NK and cytotoxic T cells, the marker gene GZMB was predominantly expressed in cluster 4, while mature B cell marker MS4A1 was most evident in cluster 5. T cell marker CD3D was highly expressed in clusters 3, 4, and 11 (**Supplementary Figure 1**).

Ultimately, these clusters were categorized as epithelial cells, APOE+ macrophages (tissue-resident), fibroblasts, CD8+ cytotoxic T cells, endothelial cells, B cells, CD4+ helper T cells, ciliated cells, dendritic cells, mesenchymal stem cells, neutrophils, mast cells, secretory cells, IFI27+ inflammatory macrophages, and CD8+ effector memory T cells (**Figure 1C**). **Figure 1D** shows the expression of STING pathway-related genes in different cell types, where XRCC5 is widely expressed across cells, particularly with the strongest expression in B cells, while PRKDC is most abundantly expressed in neutrophils.

### 3.2 STING pathway activity scoring

Cells were scored for their STING pathway activity using genes associated with the STING pathway from the Genecard database (https://www.gsea-msigdb.org/gsea/index.jsp). The results indicated that neutrophils, epithelial cells, B cells, and T cells had higher scores, suggesting active expression of STING pathway-related genes in these cells, while inflammatory macrophages showed lower scores (**Figures 1E, F**).

### 3.3 Differential cell-cell communication between high-score and low-score groups

**Figure 2:**
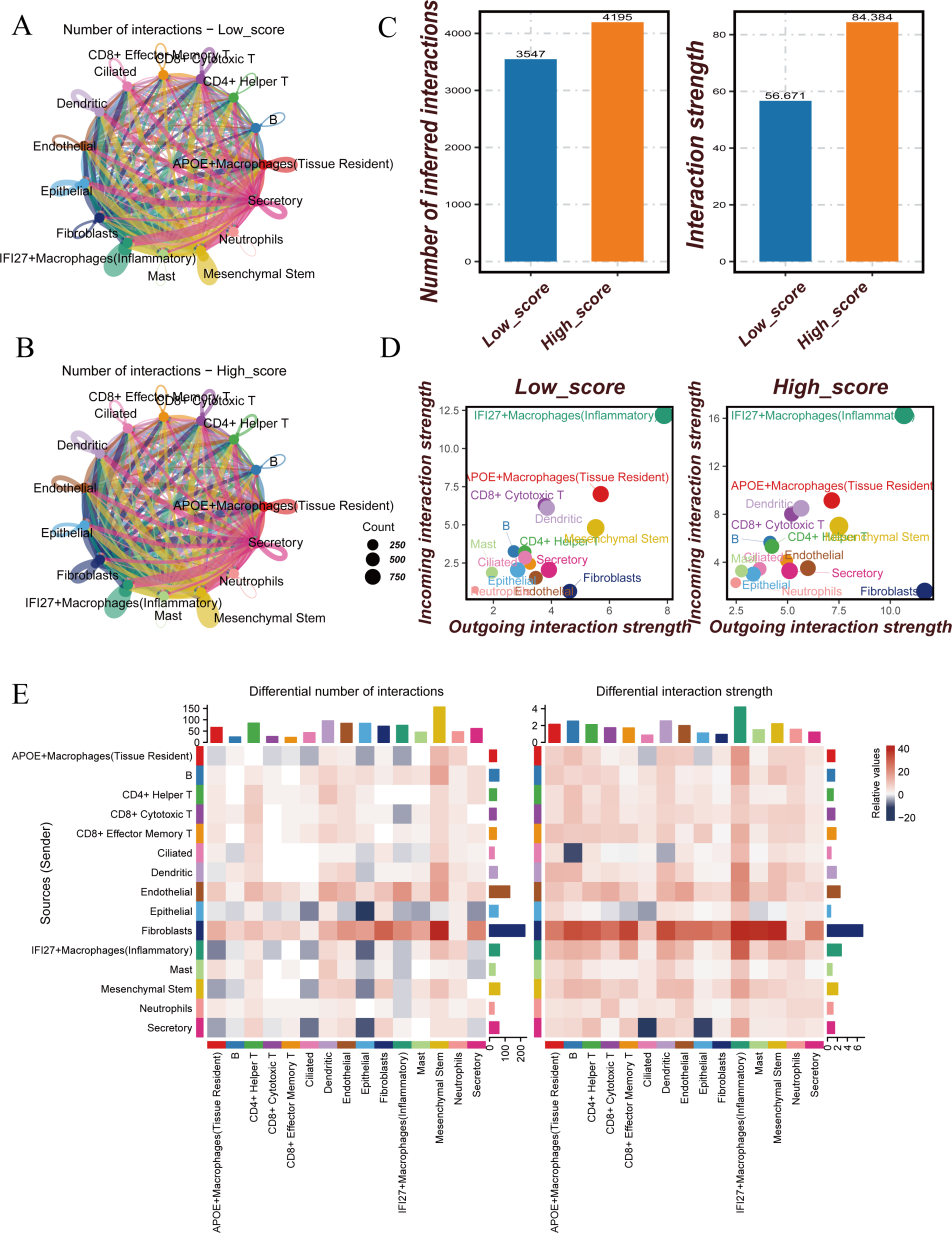
Cell-cell communication analysis between Hight-score and Low-score cells. **(A)** Number of interactions in Low-score cells. **(B)** Number of interactions in Hight-score cells. **(C)** Number of inferred interactions and interaction strength in Hight-score and Low-score cells. **(D)** Interaction strength of 15 cell clusters in Hight-score and Low-score cells. **(E)** Differences of Cell-Cell Communication.

Cells were divided into high-score (Hight-score) and low-score (Low-score) groups based on median scores. Among all cell subtypes, fibroblasts exhibited the highest outward interactions in the Hight-score cells, whereas IFI27+ inflammatory macrophages displayed the highest inward interactions. Fibroblasts had strong interactions with 14 other cell types, predominantly interacting with mesenchymal stem cells, secretory cells, APOE+ macrophages (tissue-resident), ciliated cells, and CD8+ cytotoxic T cells. These interactions were more pronounced in the Hight-score group compared to the Low-score group. Endothelial cells also showed active interactions with mesenchymal stem cells and CD4+ helper T cells (**Figures 2A, B**).

To further investigate the differences in cell-cell communication between the two groups, cell communication analysis was conducted. The results revealed that both the number and strength of cell-cell interactions in the Hight-score group were significantly higher than those in the Low-score group (**Figure 2C**). In the Hight-score group, all 15 cell subtypes displayed enhanced communication, indicating more active intercellular interactions. Both outward and inward interactions were higher in the Hight-score group compared to the Low-score group (**Figure 2D**).

A heatmap further revealed differences in communication patterns. Compared to the Low-score group, endothelial and epithelial cells in the Hight-score group exhibited enhanced interactions with other cell types, while fibroblasts showed stronger interactions with all other cell types, except for B cells (**Figure 2E**). Subsequent heatmaps displayed significantly enhanced pathways in the Hight-score group, including ANNEXIN, CLEC, CD99, LAMININ, and GRN (**Supplementary Figure 2**).

### 3.4 Transcriptional similarity and differences between high-score and low-score cells

**Figure 3:**
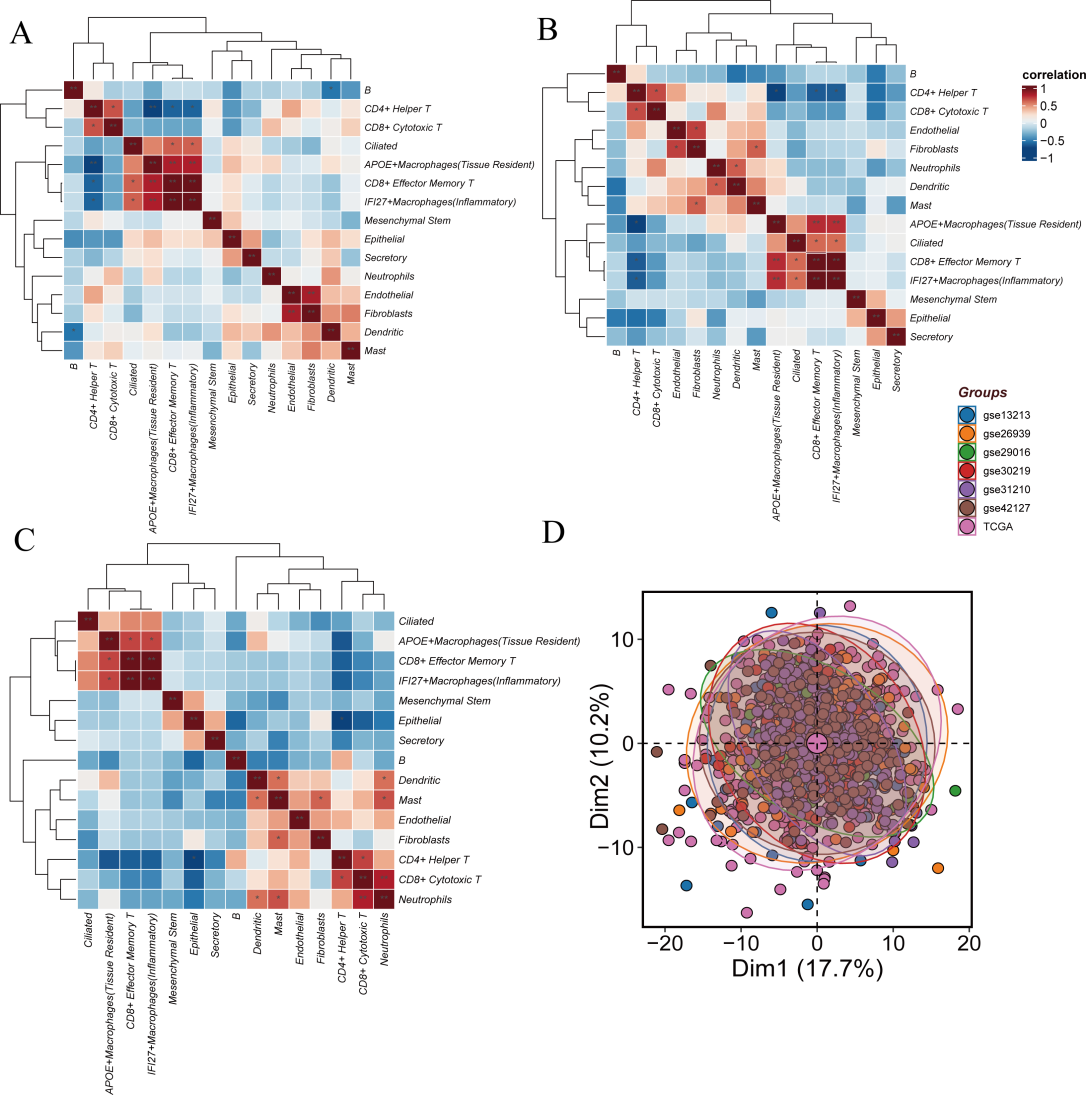
Similarities and differences between Hight-score and Low-score cells. **(A)** The difference of GSVA scores. The correlation of cell clusters in all cells. **(B)** The correlation of cell clusters in Hight-score cells. **(C)** The correlation of cell clusters in Low-score cells. **(D)** Sample distribution across datasets.

To explore the differences between Hight-score and Low-score cells, correlation analysis and clustering were performed, followed by the generation of a heatmap. The results revealed that among all cell subtypes, endothelial cells and fibroblasts, as well as CD8+ effector memory T cells and IFI27+ inflammatory macrophages, exhibited high transcriptional correlation (P<0.01). Moderate correlations were observed between CD4+ helper T cells and CD8+ cytotoxic T cells, ciliated cells and CD8+ effector memory T cells, and ciliated cells and IFI27+ inflammatory macrophages (P<0.05). No significant correlations were found between other cell types (**Figure 3A**).

In the Hight-score group, high transcriptional correlation was observed between CD8+ effector memory T cells and IFI27+ inflammatory macrophages, APOE+ tissue-resident macrophages and CD8+ effector memory T cells, as well as APOE+ tissue-resident macrophages and IFI27+ inflammatory macrophages (P<0.01). Moderate correlations were found between CD4+ helper T cells and CD8+ cytotoxic T cells, endothelial cells and fibroblasts, neutrophils and dendritic cells, fibroblasts and mast cells, ciliated cells and CD8+ effector memory T cells, and ciliated cells and IFI27+ inflammatory macrophages (P<0.05). In the Low-score cells, high correlation was observed between neutrophils and CD8+ cytotoxic T cells (P<0.01), while moderate correlations were found between APOE+ tissue-resident macrophages and IFI27+ inflammatory macrophages, APOE+ tissue-resident macrophages and CD8+ effector memory T cells, dendritic cells and mast cells, fibroblasts and mast cells, neutrophils and dendritic cells, and neutrophils and mast cells (P<0.05) (**Figures 3B, C**).

Differential gene expression between Hight-score and Low-score cells was calculated using findMarkers. To mitigate potential batch effects, seven datasets (GSE13213, GSE26939, GSE29016, GSE30219, GSE31210, GSE42127, TCGA) were batch-corrected using the ‘sva’ package. Post-correction, no significant batch effects were observed (**Figure 3D**).

### 3.5 Large-scale transcriptomic analysis and prognostic signature construction

Univariate Cox regression analysis of the TCGA dataset identified 20 prognostic genes with significant differences, including 4 genes associated with poor prognosis (ERRFI1, IL32, PPARG, and INPP4B) and 16 genes linked to favorable prognosis (such as PTPN13, EPAS1, and DOCK1) (**Supplementary Figure 3A**).

**Figure 4:**
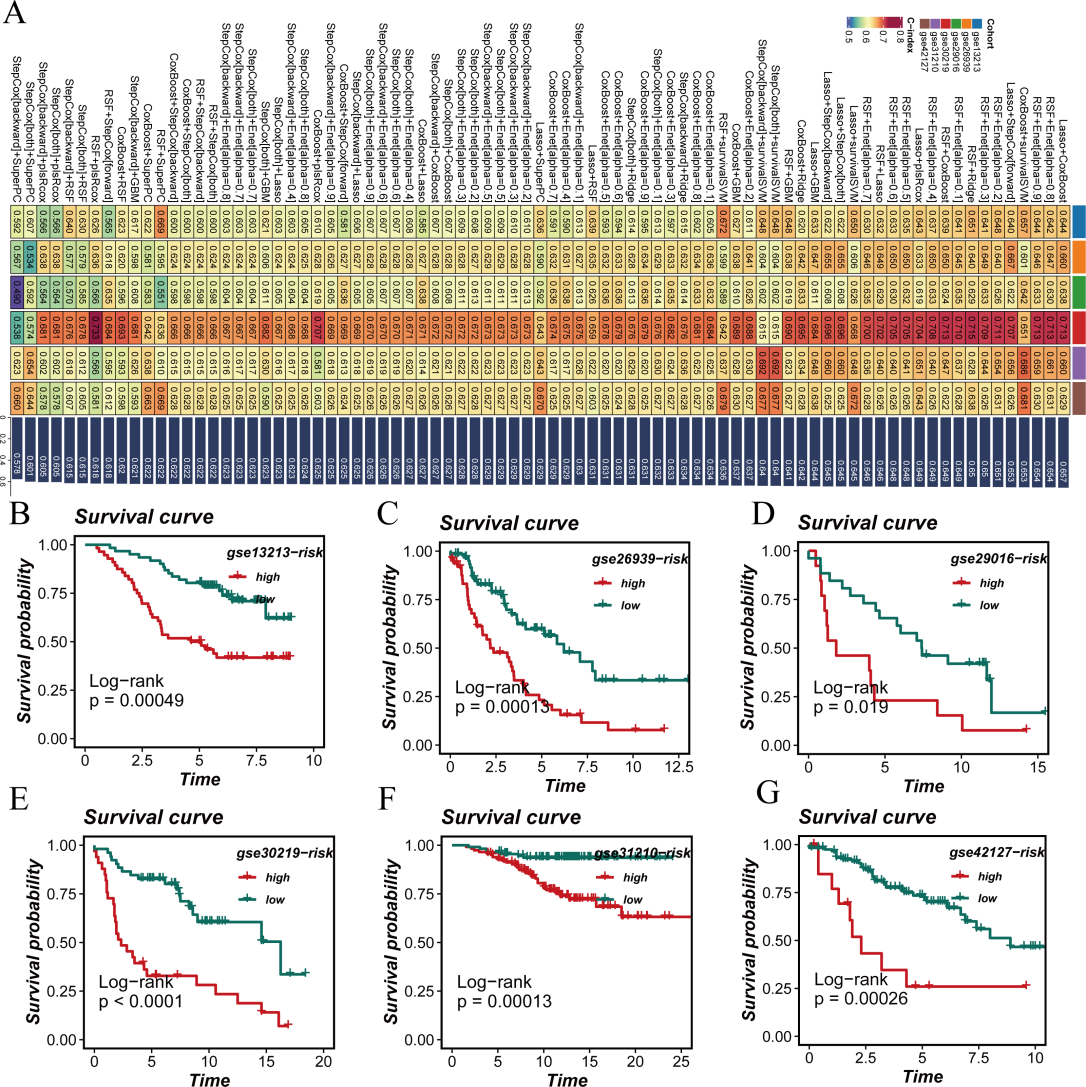
Development of a prognostic signature. **(A)** A consensus deubiquitination-related signature, by 101 machine-learning algorithms. **(B–G)** Survival curve of high-risk samples and low-risk samples across 6 datasets.

To construct the STING pathway-related signature (STINGsig), 101 machine learning algorithms were used to evaluate the 20 prognostic genes. The TCGA dataset served as the training set, while six other datasets from GEODE were used as validation sets. A 10-fold cross-validation approach was applied to construct 101 predictive models, and the C-index for both the training and validation sets was calculated (**Figure 4A**). Among these models, the Lasso+CoxBoost model performed the best, demonstrating strong performance in both the training and validation sets.

**Figure 5:**
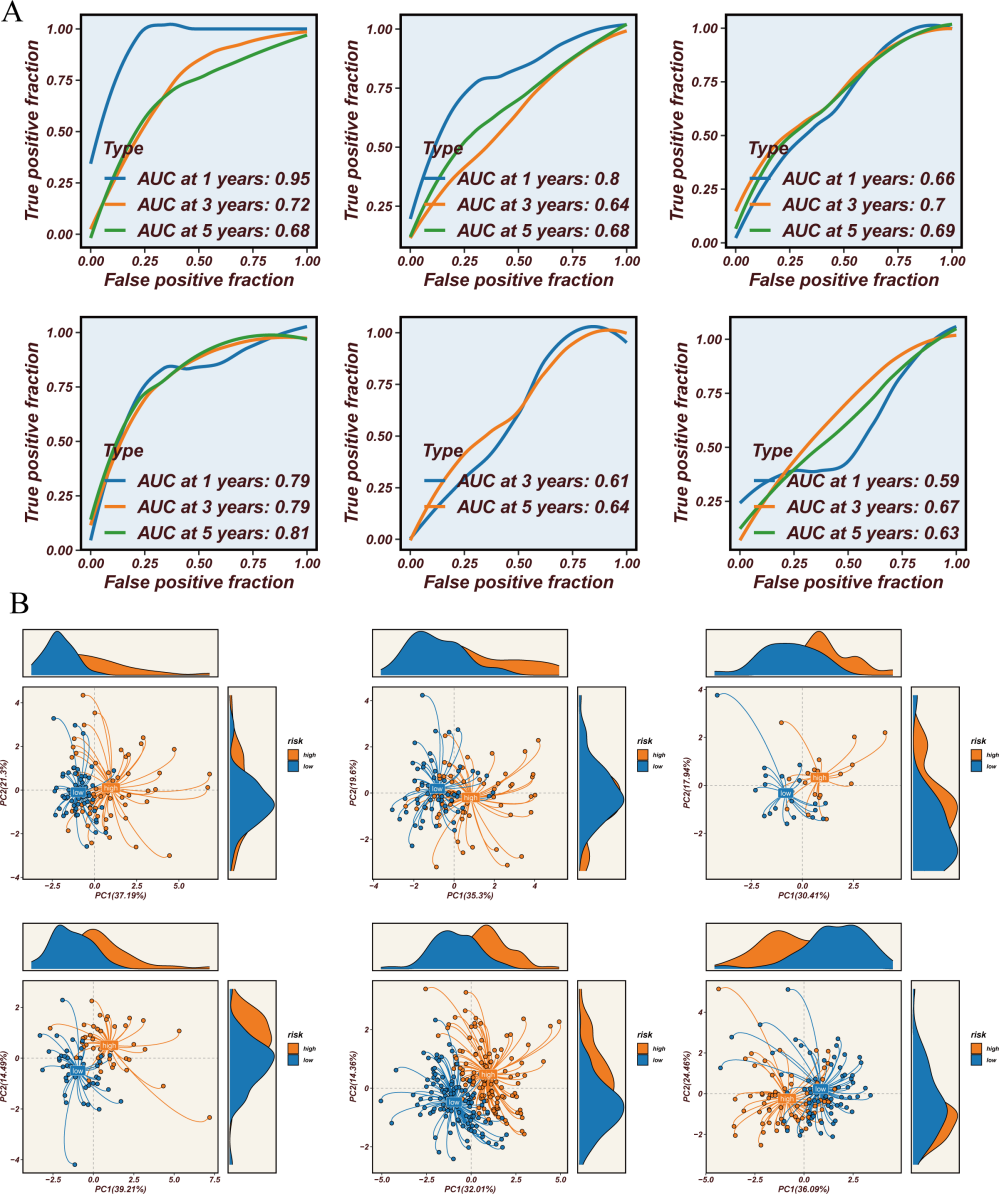
Prediction value and immune microenvironment. **(A)** ROC curve for 1-year, 3-year, and 5-year survival predictions. **(B)** PCA of high-risk samples and low-risk samples across 8 datasets.

Survival analysis across all seven datasets, using median risk scores as a threshold, showed that high-risk patients had significantly poorer prognoses compared to low-risk patients, with statistical significance (**Figures 4B–G**). The predictive performance of the model was assessed using ROC curves (**Figure 5A**). Overall, the model exhibited strong predictive capability in certain validation datasets (such as GSE13213 and GSE30219), with AUC values exceeding 0.7 for 1-year, 3-year, and 5-year survival predictions. Based on the results from all seven datasets, it can be concluded that the model has excellent prognostic predictive value.

### 3.6 Immune microenvironment and STINGsig

**Figure 6:**
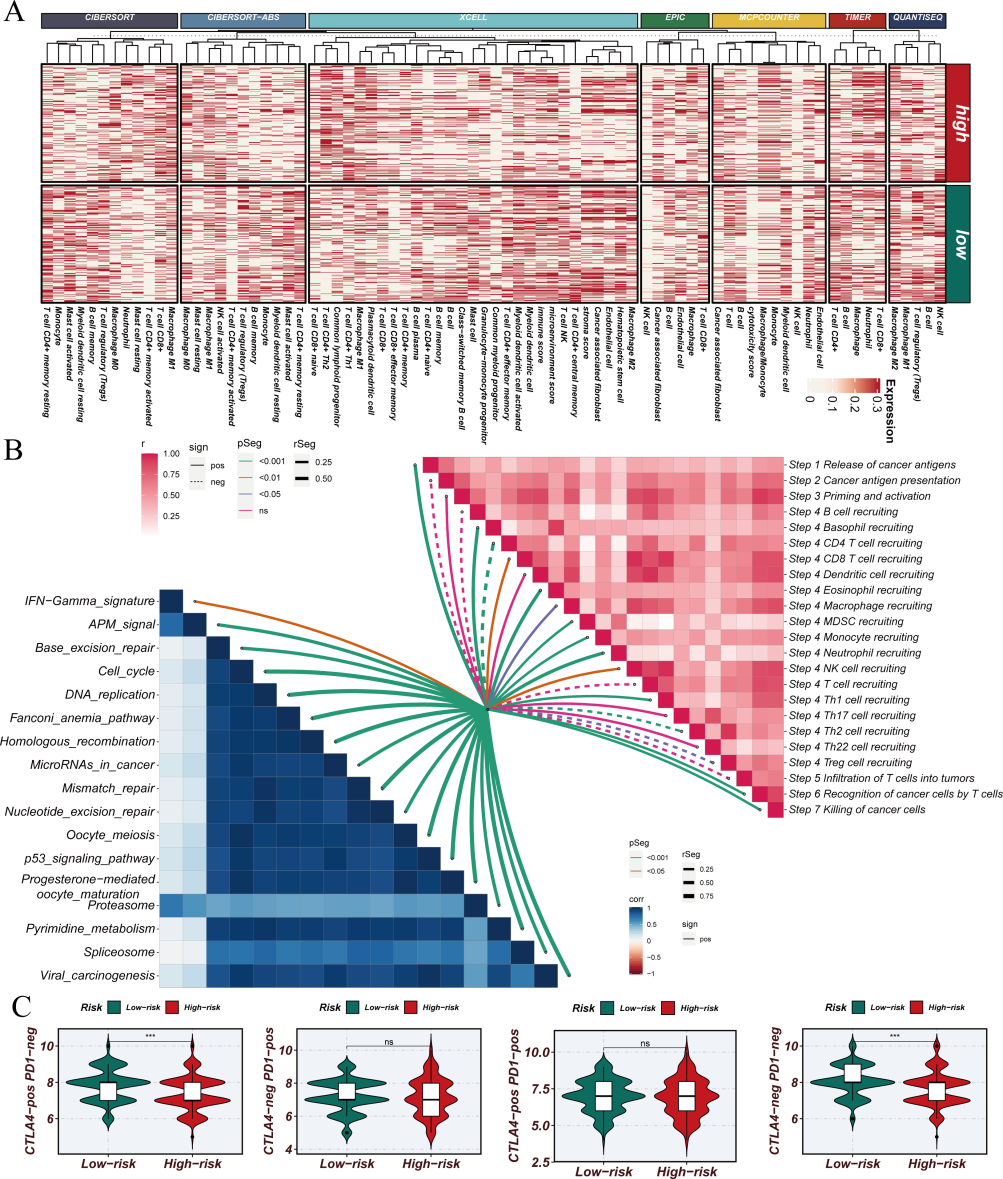
Construction and validation of STINGsig using machine learning. **(A)** Immune infiltration analysis of high-risk samples and low-risk samples. **(B)** Evaluation of the correlation between cancer immune cycle, immunotherapy pathways, and PIS using GSVA. **(C)** Prediction of IPS for TCGA-LUAD patients using The Cancer Immunome Atlas, consistently indicating higher IPS and greater sensitivity to immunotherapy in the low-risk group. LUAD, lung adenocarcinoma; IPS, immunophenoscore; TCGA, The Cancer Genome Atlas. *** indicates statistical significance with p < 0.001. NS indicates “not significant” (no statistical significance).

Using the STINGsig, samples were divided into high-risk and lowrisk groups, and PCA analysis was conducted to observe expression differences. The high-risk and low-risk groups were distinctly separated in datasets such as GSE13213, GSE31210, and GSE42127 (**Figure 5B**). To explore differences in the immune microenvironment between high and low-risk groups, seven immune infiltration algorithms from the TCGA dataset were employed (CIBERSORT, CIBERSORT-ABS, XCELL, EPIC, MCPCOUNTER, TIMER, and QUANTISEQ) (**Figure 6A**). The results indicated that cancer-associated fibroblasts, neutrophils, plasmacytoid dendritic cells (pDCs), and CD4+ Th1 and Th2 T cells were more abundant in high-risk samples, while NK cells, M2 macrophages, B cells, CD4+ T cells, CD8+ T cells, CD4+ central memory T cells, CD4+ memory resting cells, and activated mast cells were significantly increased in the low-risk group. Furthermore, correlation analysis of STINGsig with cancer-related immune therapeutic pathways and immune cycle steps (**Figure 6B**) revealed its functional mechanism: STINGsig showed significant positive correlations with cell proliferation pathways such as cell cycle and DNA repair, while negatively correlating with cancer antigen release and B cell and T cell recruitment, reflecting its complex role in tumor immune regulation. Based on TCIA analysis, the low-risk group demonstrated significantly higher immunotherapy scores than the high-risk group (**Figure 6C**), suggesting that low-risk patients possess immune characteristics more favorable for immunotherapy. These findings further illustrate the important regulatory role of STINGsig in the immune microenvironment and immunotherapy, providing new evidence for optimizing immunotherapy strategies.

### 3.7 ERRFI1 loss enhances anti-tumor immunity and synergizes with α-PD1 therapy in lung cancer model

**Figure 7:**
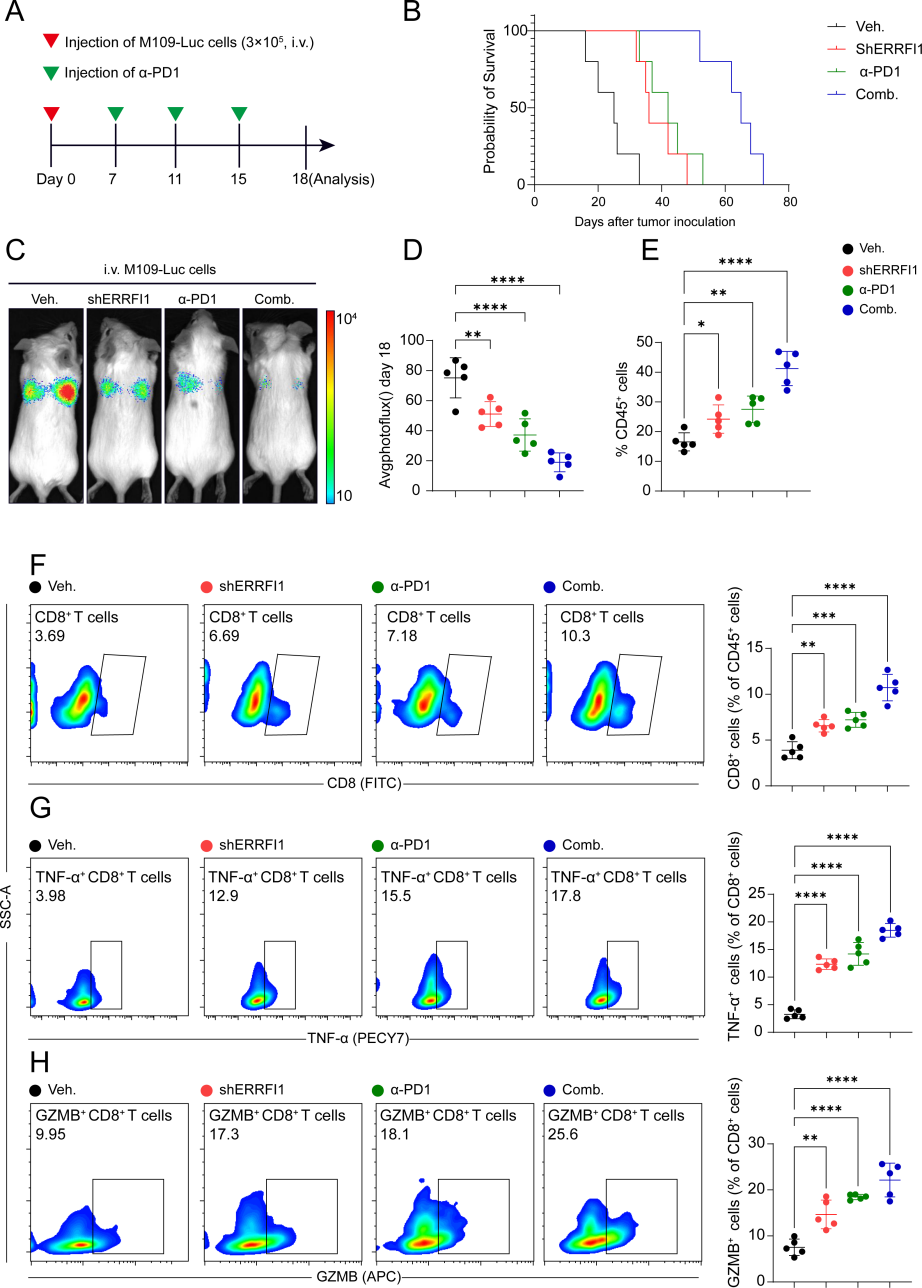
ERRFI1 loss enhances anti-tumor immunity and synergizes with α-PD1 therapy in lung cancer model. **(A)** Schematic of the experimental design. Mice were intravenously injected with M109-Luc cells (3×10^5) on day 0, followed by treatments (Veh., shERRFI1, α-PD1, or Comb.) from day 7 to day 15. Analysis was performed on day 18. **(B)** Kaplan-Meier survival curves show enhanced survival in mice treated with shERRFI1, α-PD1, or the combination, with the greatest survival benefit observed in the combination group. **(C, D)** Tumor burden assessed by bioluminescence imaging (photoflux) on day 18. The combination of shERRFI1 and α-PD1 significantly reduced tumor burden compared to individual treatments or vehicle control (****P < 0.0001). **(E–H)** Flow cytometry analysis of immune cell infiltration in the tumor microenvironment. **(E)** Percentage of CD45+ leukocytes. **(F)** Percentage of CD8+ T cells among CD45+ cells. **(G)** Percentage of TNF-α+ cells among CD8+ T cells. **(H)** Percentage of GZMB+ cells among CD8+ T cells. * p < 0.05, ** p < 0.01, *** p < 0.001, **** p < 0.0001.

Subsequently, we conducted a more in-depth analysis of ERRFI1, a key gene within the STING signature. In a lung cancer model, the combination of ERRFI1 knockdown (shERRFI1) and anti-PD1 (α-PD1) therapy significantly reduced tumor burden (p < 0.0001) and improved survival rates, demonstrating a synergistic effect compared to monotherapy or the control group (**Figures 7A–D**). Flow cytometry analysis revealed that the combination treatment significantly enhanced the infiltration of CD45+ leukocytes (p < 0.05, **Figure 7E**) and markedly increased the proportion of CD8+ T cells among CD45+ cells (p < 0.001, **Figure 7F**). Furthermore, the combination therapy significantly elevated the frequencies of TNF-α+ and GZMB+ CD8+ T cells (p < 0.0001, **Figures 7G, H**), indicating enhanced activation of cytotoxic T cells. These findings suggest that the loss of ERRFI1 potentiates antitumor immunity and synergizes with α-PD1 therapy to promote tumor control.

## 4 Discussion

Lung cancer are among the most common forms of cancer and are one of the leading causes of cancer-related mortality worldwide (38, 39). The majority of patients are diagnosed at advanced stages with metastasis, resulting in a poor prognosis (40). While immune checkpoint inhibitors have revolutionized lung cancer treatment, only a subset of patients responds to these therapies. This study identifies differential activity in the STING signaling pathway, revealing significant heterogeneity in lung cancer and its potential impact on immunotherapy response. The STING pathway, as a crucial mediator of innate immunity and T cell priming, plays a vital role in antitumor immune responses. Notably, high STING scores are associated with enhanced tumor-promoting pathways, active immune interactions, and enrichment in fibroblasts and IFI27+ inflammatory macrophages. In contrast, low scores correlate with an epithelial phenotype and reduced immune activity. The prognostic risk signature based on this scoring model demonstrates clinical predictive value and potential utility in patient stratification for immunotherapy.

Consistent with previous research, this study successfully identified 15 cell types, including epithelial cells, APOE+ macrophages (tissue-resident), fibroblasts, CD8+ cytotoxic T cells, endothelial cells, B cells, CD4+ helper T cells, ciliated cells, dendritic cells, mesenchymal stem cells, neutrophils, mast cells, secretory cells, IFI27+ inflammatory macrophages, and CD8+ effector memory T cells. The expression variations of these cell types across populations provide a solid foundation for subsequent analysis (41–43). Furthermore, the study reveals that pathways associated with tumor cell growth, metastasis, immune suppression, and immune evasion are upregulated in the high-score group. These pathways are closely linked with various immune cells, including T cells, B cells, dendritic cells, and NK cells (44–47). This suggests that the high-score group exists in an immune-suppressive microenvironment, indicating that the STING signaling pathway may be related to immune evasion and immune killing mechanisms in lung malignancies, making it a potential therapeutic target for the disease. Intercellular interaction analysis shows that both the quantity and intensity of cell interactions are enhanced in the high-score group, suggesting that these cells exhibit more active intercellular communication, and that the STING pathway may be involved in augmented cell-to-cell communication.

Through univariate Cox analysis, the study identifies 20 prognostic genes with significant differences, involving critical cellular processes such as immune response, cell proliferation, migration, apoptosis, metabolism, transcriptional regulation, and signal transduction. Among these genes, ERRFI1 (EGFR Repressor Protein 1) functions as a negative regulator in cellular signaling (48). As a tumor suppressor, ERRFI1 directly interacts with EGFR to inhibit its activation, thereby suppressing tumor cell proliferation and metastasis (49). In this study, ERRFI1 is negatively correlated with prognosis and holds potential as a target for tumor immunotherapy, warranting further investigation. Interleukin-32 (IL-32), a pro-inflammatory cytokine produced by various immune cells, plays a pivotal role in the tumor immune microenvironment by promoting tumor-related inflammatory responses, enhancing tumor cell survival, migration, and invasiveness, and aiding immune evasion through the production of immunosuppressive cytokines (50, 51). PTPN13 (also known as FAP-1, Fas-associated phosphatase-1), a non-receptor protein tyrosine phosphatase, primarily inhibits pro-tumor signaling pathways such as PI3K/Akt and MAPK, thereby suppressing tumor cell proliferation and survival (52–54). Endothelial PAS domain-containing protein 1 (EPAS1), a member of the hypoxia-inducible factor (HIF) family, has been shown to promote proliferation, invasion, and migration in HeLa and SiHa cells (55). However, in thyroid malignancies, EPAS1 acts as a tumor suppressor (56). In this study, EPAS1 is also associated with a favorable prognosis, highlighting its potential clinical significance. Dedicator of Cytokinesis 1 (DOCK1), an important GTPase-activating protein, influences T cell migration, activation, and immune response by regulating the small GTPase Rac1 (57). In this study, DOCK1 is associated with a favorable prognosis, suggesting that T cell activation may enhance immune killing in LUAD. However, the role of T cells in tumorigenesis remains controversial, thus warranting further investigation. This underscores the necessity for a deeper exploration of immune functions in lung malignancies.

The STINGsig demonstrated robust predictive performance and serves as a valuable tool for assessing patient prognosis in lung malignancies, providing powerful insights for personalized treatment planning. This signature is particularly relevant in the context of immunotherapy, as it reflects the underlying immune status of the tumor microenvironment. Furthermore, it can aid in identifying high-risk patients, enabling the development of tailored therapeutic strategies to optimize clinical decision-making and improve overall survival rates. Our *in vivo* experiments demonstrated that knockdown of ERRFI1, a critical gene within the STING signature, significantly enhances antitumor immunity and synergizes with anti-PD1 therapy in a lung cancer model. Compared to monotherapy or vehicle control, the combination of shERRFI1 and anti-PD1 treatment markedly reduced tumor burden in mice. This synergistic effect likely occurs through the modulation of both innate and adaptive immune responses, as the STING pathway activation can enhance dendritic cell function and subsequent T cell priming. Kaplan-Meier survival analysis revealed that the combination therapy conferred the greatest survival benefit, significantly prolonging survival compared to either monotherapy or control groups. Moreover, the combination treatment significantly elevated the frequencies of TNF-α+ and GZMB+ CD8+ T cells, indicating enhanced activation of cytotoxic T cells and improved antitumor immune responses. These findings underscore the pivotal role of ERRFI1 in modulating antitumor immunity and highlight its potential as a therapeutic target to augment the efficacy of immune checkpoint blockade. The observed synergy between ERRFI1 inhibition and anti-PD1 therapy suggests a promising strategy for improving immunotherapy outcomes in lung cancer patients.

## 5 Conclusions

This study integrates single-cell RNA sequencing data and large-scale transcriptomic datasets to systematically explore the role of the STING signaling pathway in intercellular communication and immune mechanisms. Through biological analysis, this study enhances our understanding of the STING pathway in LUAD, offering new perspectives on tumor immune microenvironments and laying the groundwork for potential applications in tumor diagnosis, therapy, and personalized treatment. Despite its scientific value and potential applications, the study has certain limitations. Firstly, due to cost and sample availability constraints, this research relies on public datasets rather than proprietary samples, which may limit the accuracy of the results in certain populations. Secondly, experimental validation was not possible, as some conclusions are based solely on computational analyses. Furthermore, *in vivo* experiments demonstrated that the loss of ERRFI1, a pivotal gene within the STING signature, significantly enhanced antitumor immunity and exhibited a synergistic effect with α-PD1 therapy to promote tumor control. Nonetheless, the STING pathway-related signature (STINGsig) constructed in this study holds significant prognostic value, identifying key genes associated with the immune microenvironment, and offering valuable insights for the diagnosis and treatment of LUAD.

## Data Availability

This study used public data from online databases. The data can be obtained freely from TCGA (https://www.cancer.gov/ccg/research/genome-sequencing/tcga) and GEO (https://www.ncbi.nlm.nih.gov/geo/).

## Supplementary material

Supplementary Figure 1 The expression of 18 marker genes in cell clusters.

Supplementary Figure 2 Cellular pathways showing differences between low and high STING pathway scores.

Supplementary Figure 3
**(A)** Univariate COX regression analysis of STING Pathway genes. **(B)** Survival curve of high-risk samples and low-risk samples across TCGA. **(C)** ROC curve for 1-year, 3-year, and 5-year survival predictions. **(D)** PCA of high-risk samples and low-risk samples.
