# Corrigendum: Harnessing single-cell and multi-omics insights: STING pathway-based predictive signature for immunotherapy response in lung adenocarcinoma

**DOI:** 10.3389/fimmu.2025.1613095

**Published:** 2025-04-30

**Authors:** Yang Ding, Dingli Wang, Dali Yan, Jun Fan, Zongli Ding, Lei Xue

**Affiliations:** ^1^ Department of Pathology, Nanjing Drum Tower Hospital Group Suqian Hospital, Suqian, China; ^2^ Department of Lung Cancer, Tianjin Lung Cancer Center, National Clinical Research Center for Cancer, Key Laboratory of Cancer Prevention and Therapy, Tianjin’s Clinical Research Center for Cancer, Tianjin Medical University Cancer Institute and Hospital, Tianjin, China; ^3^ Department of Oncology, The Affiliated Huai’an Hospital of Xuzhou Medical University and the Second People’s Hospital of Huai’an, Huai’an, China; ^4^ Department of Thoracic Surgery, The First Affiliated Hospital with Nanjing Medical University, Nanjing, China; ^5^ Department of Geriatrics, The Affiliated Huai’an Hospital of Xuzhou Medical University and the Second People’s Hospital of Huai’an, Huai’an, China

**Keywords:** non-small cell lung cancer, lung adenocarcinoma, single-cell RNA sequencing, prognosis signature, machine learning

In the original article, there was an error in the author list. The first author’s name was incorrectly listed as “Ding Yang” instead of the correct name “Yang Ding”.

The authors apologize for this error and state that this does not change the scientific conclusions of the article in any way.

