# Supplementary figures and images for: Harnessing single-cell and multi-omics insights: STING pathway-based predictive signature for immunotherapy response in lung adenocarcinoma
Source: Front Immunol. 2025 Apr 16;16:1575084. doi: 10.3389/fimmu.2025.1575084 (PMC12040650; doi:10.3389/fimmu.2025.1575084)

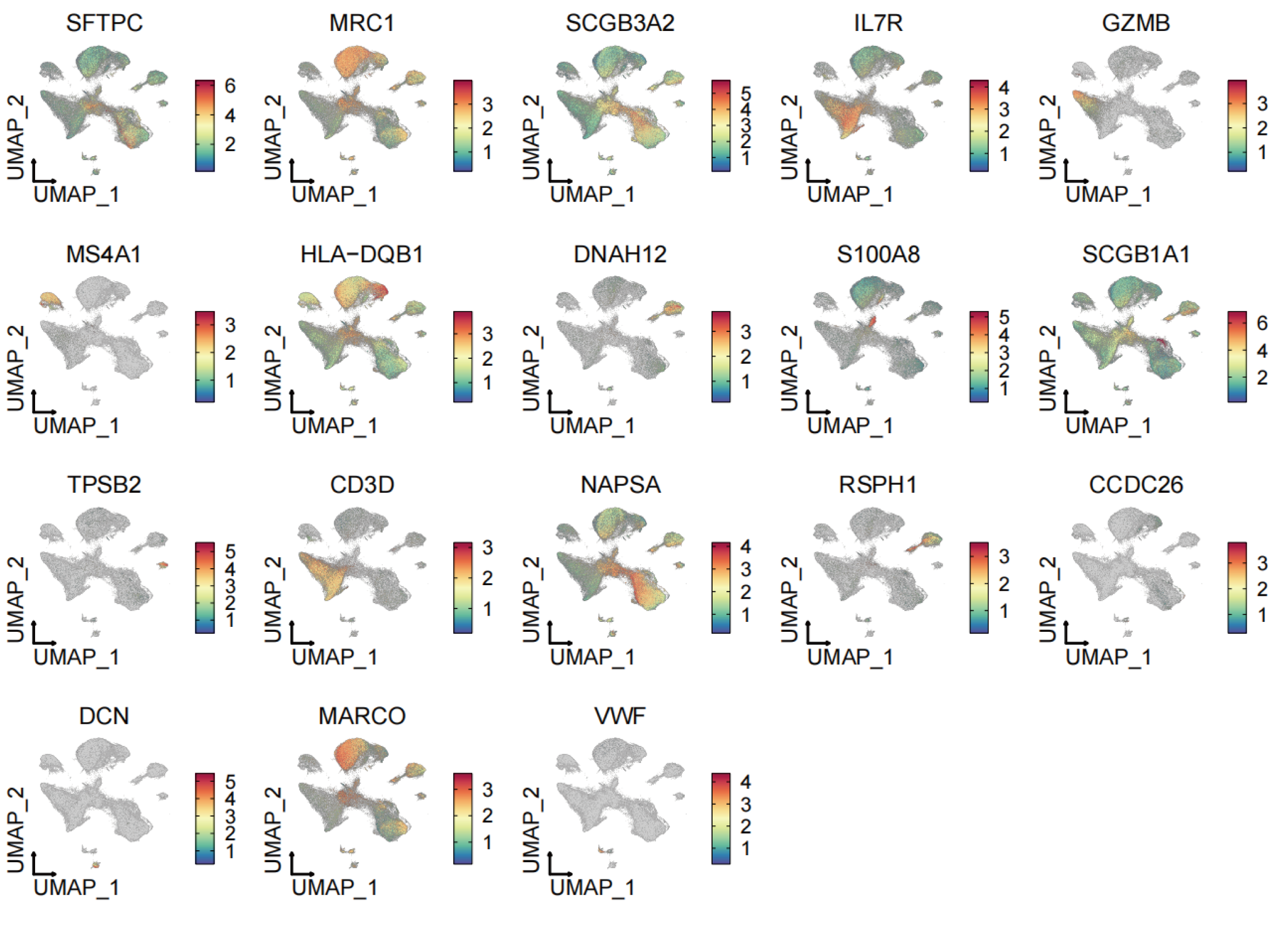

Supplement: Supplementary Figure 1 — The expression of 18 marker genes in cell clusters. [file Image1.tif]

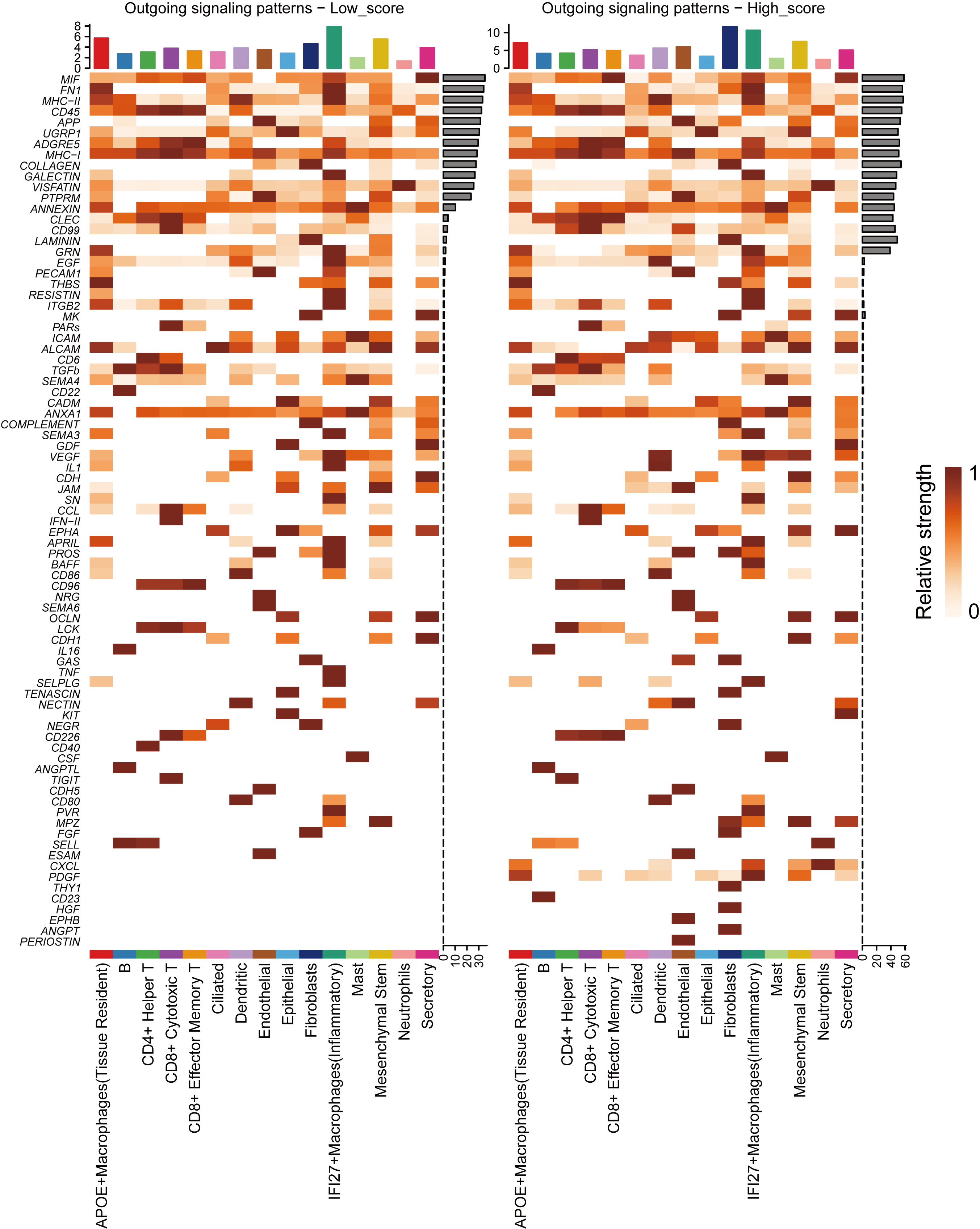

Supplement: Supplementary Figure 2 — Cellular pathways showing differences between low and high STING pathway scores. [file Image2.tif]

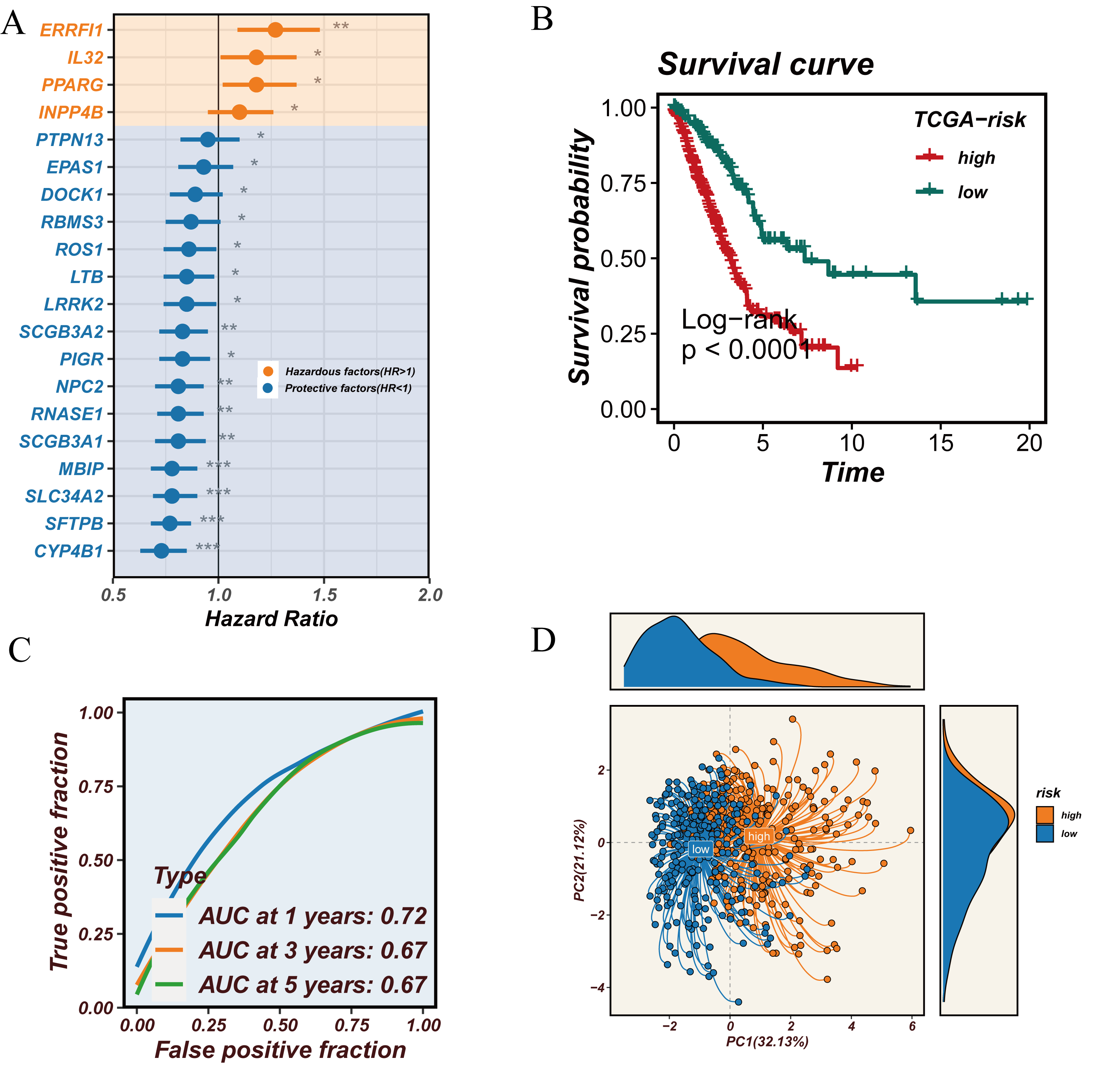

Supplement: Supplementary Figure 3 — (A) Univariate COX regression analysis of STING Pathway genes. (B) Survival curve of high-risk samples and low-risk samples across TCGA. (C) ROC curve for 1-year, 3-year, and 5-year survival predictions. (D) PCA of high-risk samples and low-risk samples. [file Image3.tif]
